# Instrumental learning enhances the intrinsic excitability of basal amygdala projection neurons

**DOI:** 10.1101/lm.054161.125

**Published:** 2026-01

**Authors:** Eddie T. Wise, Philip Jean-Richard-dit-Bressel, Joanna O-Y. Yau, Gavan P. McNally, John M. Power

**Affiliations:** 1 Translational Neuroscience Facility and Department of Physiology, School of Biomedical Sciences, UNSW Sydney, Sydney NSW 2052, Australia; 2 School of Psychology, UNSW Sydney, Sydney NSW 2052, Australia

## Abstract

The basolateral amygdala (BLA) is critical for Pavlovian and instrumental emotional association learning. Pavlovian fear conditioning is accompanied by increased excitability of BLA neurons. Here we tested whether instrumental learning similarly enhances BLA excitability. Electrophysiological recordings were taken from BLA neurons in brain slices prepared from instrumentally trained Long–Evans rats. Both reward and punishment training increased intrinsic excitability. Moreover, excitability positively correlated with performance on the punishment task, suggesting a functional link between neuronal excitability and learning. These findings support the idea that enhanced excitability facilitates synaptic plasticity and circuit integration during instrumental learning.

An essential component of adaptive behavior is the ability to evaluate the consequences of one's actions. Through experience, animals and humans learn to associate specific behaviors with rewarding or punishing outcomes, allowing them to modify future behavior to obtain reward and avoid punishment. This instrumental learning is critical for flexible, goal-directed behavior and relies on the ability to form and store emotional associations (Ostlund and Balleine 2008) and may be disrupted in disorders such as conduct disorder and addiction that can involve maladaptive responses to adverse consequences (McNally et al. 2023). The basolateral amygdala (BLA) is critical for forming emotional associations (LaBar et al. 1998; Gottfried et al. 2002; Wassum and Izquierdo 2015; Jean-Richard-Dit-Bressel et al. 2018). While instrumental learning has clinical relevance, our knowledge of how the amygdala encodes emotional associations is largely restricted to Pavlovian conditioning.

Pavlovian conditioning is associated with several long-term neurophysiological changes to BLA circuits including long-term enhancement of excitatory inputs (Lynch 2004), decreased local circuit inhibition (Lucas et al. 2016), and enhanced intrinsic excitability in glutamatergic projection neurons (Motanis et al. 2014; Sehgal et al. 2014, 2023). While synaptic plasticity has long been the focus of most studies, there is growing recognition that intrinsic neuronal excitability, the likelihood of firing in response to input, plays a vital role in memory formation (Schulz 2006; Sehgal et al. 2018). Changes in intrinsic excitability influence how neurons integrate synaptic signals and modulate the induction of synaptic plasticity (Sah and Bekkers 1996; Power et al. 2011). Neurons with elevated excitability are more likely to be incorporated into memory traces (Josselyn and Frankland 2018; Josselyn and Tonegawa 2020). Here, we examined whether instrumental training with either appetitive (reward) or aversive (punishment) outcomes alters the intrinsic excitability of BLA glutamatergic projection neurons.

Male and female Long–Evans rats (2–4 months old) were obtained from colonies maintained by the School of Psychology (UNSW). Rats were housed in groups of two to four and kept on a 12:12 light–dark cycle (lights on at 7:00). All procedures were approved by the UNSW Animal Care and Ethics Committee and performed in accordance with the Animal Research Act 1985 (NSW), under the guidelines of the National Health and Medical Research Council Code for the Care and Use of Animals for Scientific Purposes in Australia (2013).

Rats were food-restricted for 1 day in their home cages before training. Instrumental conditioning was performed as previously described (Fig. 1A; Jean-Richard-Dit-Bressel et al. 2022). Each rat received one daily session of lever press acquisition for food reward, while naive controls were given supplemental food to match the amount earned by trained animals. Body weights were maintained at 90% of baseline, though individual food intake was not tracked. Water was available ad libitum. Training began with two 1 h magazine sessions in which both levers (left and right) were presented simultaneously and reinforced on a fixed ratio-1 (FR-1) schedule. In subsequent sessions, levers were presented alternately for 5 min blocks across a 40 min session, totaling eight trials (four per lever), with reinforcement on a variable interval 30 sec (VI30) schedule. This training continued for 7 days with pressing increasing across days (*F*_(1,18)_ = 81.2, *P* < 0.001), indicating successful instrumental reward learning (Fig. 1B–D). The rate of responding in the to-be punished and to-be reward group appeared to diverge toward the end of lever press training. As animals were randomly allocated to these groups, the reason is unclear. Unexpectedly, rates of responding were slightly higher on R2 than on R1 (*F*_(1,18)_ = 6.89, *P* = 0.017); however, the increase in the rate of responding between the two levers did not differ across days (i.e., no group main effect or group × day interaction) (*F*_(1,18)_ = 0.296, *P* = 0.59).

**Figure 1.**
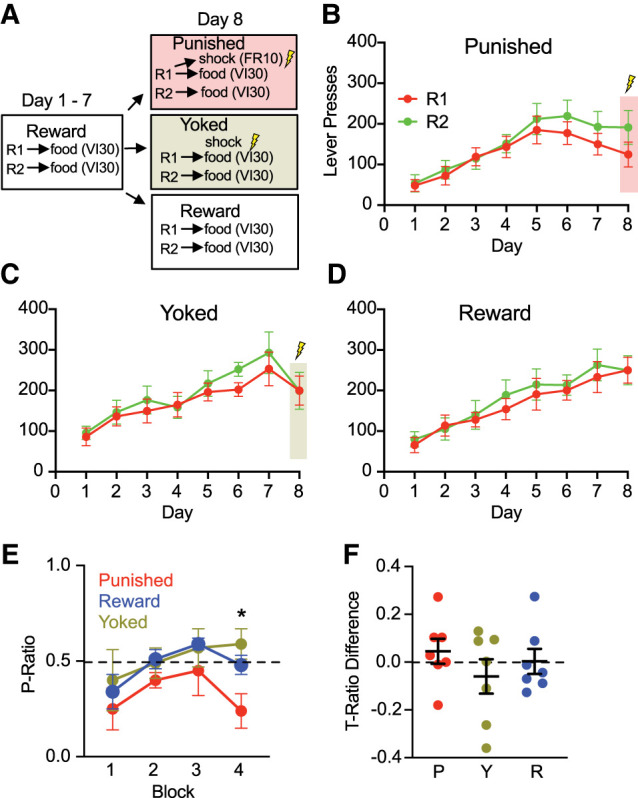
Behavioral data. (*A*) Experimental design. (*B*–*D*) Lever presses for groups across days 1–8. (*E*) Day 8 preference ratios (*P*-ratio) across four 5 min time blocks; block 4 *P*-ratio was reduced in Punished animals. (*F*) Overall *T*-ratio differences (preference change from day 7 to day 8) for Punished (P), Yoked (Y), and Reward (R). (*) *P* < 0.05.

On day 8, rats were randomly assigned to one of three groups. “Punished” rats continued VI30 training, but responses on one lever (R1) also delivered a 0.5 sec, 0.4 mA footshock on an FR10 schedule, independent of pellet delivery. Responses on the other lever (R2) remained unpunished. Each punished rat was paired with a “Yoked” rat that received the same number and timing of shocks, but noncontingently, modeling Pavlovian fear conditioning. “Reward” rats received an additional VI30 session without shock. To assess learning-related suppression, a preference ratio (*P*-ratio)—the proportion of responses on the punished lever—was calculated across 5 min blocks. With training, Punished rats showed selective suppression of R1 (*P*-ratio <0.5), with group differences emerging in the final block (*F*_(2,17)_ = 5.61, *P* = 0.01; Fig. 1E). Punished rats showed a greater preference for the unpunished lever than Yoked and Reward rats (*F*_(1,17)_ = 10.0, *P* < 0.05), indicating punishment learning. Yoked and Reward rats did not differ (*F*_(1,17)_ = 1.18, *P* > 0.05). To account for lever preferences, training suppression ratios (*T*-ratios) were calculated for each lever (*T* = D8/[D7 + D8]) such that lever pressing on the final session (D8) was measured relative to the previous session (D7). A positive difference between *T*-ratios for unpunished and punished levers (*T*_(unpun)_−*T*_(pun)_) > 0 reflects a bias away from the punished lever (Jean-Richard-Dit-Bressel et al. 2019). Although five of seven Punished rats showed positive *T*-ratio differences, group-level effects were not significant (*F*_(2,18)_ = 0.79, *P* = 0.47), likely due to the delayed emergence of suppression within the session (Fig. 1F).

Intrinsic excitability was assessed after the final session. Coronal brain slices (300 µm) were prepared 1 h following the final behavioral session according to standard methods (Yau et al. 2021). Slices were transferred to a recording chamber continuously perfused with oxygenated standard artificial cerebral spinal fluid (30°C), and whole-cell patch-clamp recordings were made from BLA neurons as described previously (Yau et al. 2021). Recordings were restricted to electrophysiologically phenotyped glutamatergic projection neurons located in caudal regions of the basal nucleus, which is implicated in punishment (Fig. 2A; Jean-Richard-Dit-Bressel and McNally 2015). Glutamatergic projection neurons were distinguished from GABAergic neurons based on their action potential (AP) half-width (>0.7 msec), their small fast afterhyperpolarization (<15 mV), and frequency-dependent AP broadening (Sah et al. 2003; Power et al. 2011). Cells with resting membrane potentials (RMP) < −55 mV, AP amplitudes > 50 mV, and membrane resistances (Rm) > 60 MΩ were considered healthy and included in the data set.

**Figure 2.**
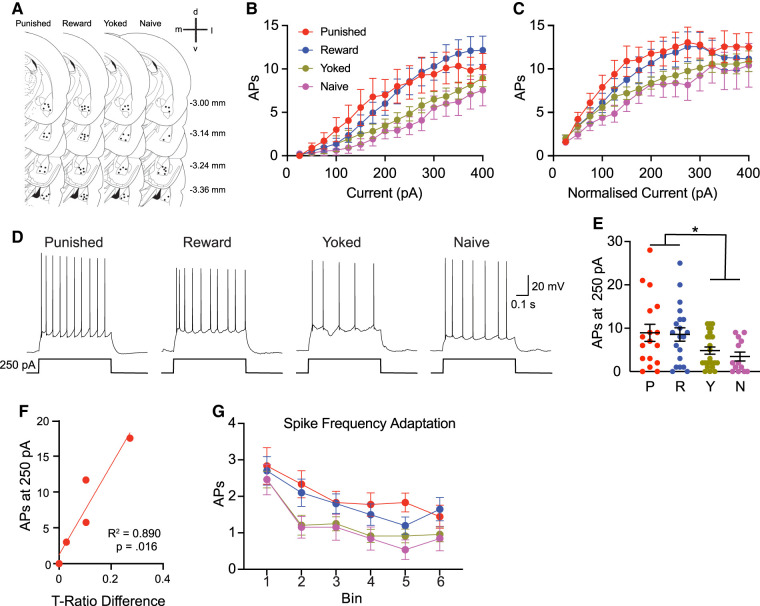
Learning-associated intrinsic excitability changes. Anatomical distribution of recorded BLA neurons (*A*). Estimated distance from bregma is shown to the *right*. APs as a function of raw current injection for neurons from Punished (P), Reward (R), Yoked (Y), and Naive (N) rats (*B*). APs normalized to rheobase current, with increasing firing at higher current amplitudes (*C*). Spikes evoked by 250 pA, neurons from showing that Yoked and Naive animals had reduced firing compared to Punished and Reward (*D*,*E*). Regressions performed between *T*-ratio differences and mean spikes evoked by 250 pA mean per rat from firing properties data for Punished rats (*F*); BLA excitability predicted behavioral bias toward the unpunished lever for Punished rats. Number of APs evoked by a 600 msec current injection (100 pA above rheobase) plotted at 100 msec intervals (*G*). (*) *P* < 0.05.

To assess if training altered intrinsic excitability, neurons were maintained at −65 mV and subjected to a series of 600 msec depolarizing current injections (0 to +400 pA, 25 pA increments; Fig. 2B). Two-way repeated measures ANOVA revealed an increase in AP firing with increasing current (*F*_(14_,_994)_ = 60.0, *P* < 0.0001), a significant interaction between current and behavior group (*F*_(42, 994)_ = 1.7, *P* = 0.0028), but no main effect of behavioral group (*F*_(3,70)_ = 2.7, *P* > 0.05). Similar effects were observed relative to rheobase; a current-dependent increase in AP firing (*F*_(19,1331)_ = 40.1, *P* < 0.0001), a significant interaction between current and behavior group (*F*_(21,493)_ = 1.8, *P* = 0.013; Fig. 2C), and no behavior group effect (*F*_(3,70)_ = 1.37, *P* = 0.26). To further probe excitability, we compared the number of APs evoked by a 250 pA current step (Fig. 2D,E), to avoid spike count floor and ceiling effects. Spike counts differed across groups (*F*_(3,70)_ = 3.49, *P* = 0.02), with Punished and Reward firing more APs than Yoked and Naive (*F*_(1,70)_ = 10.5, *P* < 0.05). No differences were found between Punished and Reward (*F*_(1,70)_ = 0.04, *P*  > 0.05) or Yoked and Naive (*F*_(1,70)_ = 0.45, *P* > 0.05). Despite the apparent behavioral differences, there were no significant group differences in behavioral performance, and excluding subjects that showed a 15% reduction in pressing of either lever between days 6 and 7 yields comparable results, with group differences in number of APs evoked at 250 pA (*F*_(3,58)_ = 3.67, *P* = 0.017); Punished and Reward fired more APs than Yoked and Naive groups (*F*_(1,58)_ = 9.37, *P* < 0.05). Together, these findings show two things: (1) instrumental training (Punishment or Reward) enhances BLA excitability and (2) noncontingent shock delivery reverts excitability to the Naive state.

Since Punished and Reward groups did not differ, it remains unclear whether these changes specifically reflect punishment learning or general instrumental contingencies, as both groups received pellets for lever pressing. To explore this, we examined whether BLA excitability predicted behavioral bias between levers (Fig. 2F). Linear regressions were performed comparing each rat's mean spike count at 250 pA with their lever press training ratio difference. No significant correlations were found in the Punished (*R*^2^ = 0.246, *P* = 0.26), Yoked (*R*^2^ = 0.0027, *P* = 0.91), or Reward group (*R*^2^ = 0.0001, *P* = 0.98). Within the Punished group, animals that exhibited a behavioral bias (*T*-ratio difference >0) showed a strong positive correlation between excitability and bias (*R*^2^ = 0.890, *P* = 0.016; *n* = 5), suggesting that increased BLA excitability may support punishment learning in behaviorally responsive individuals. The relationship between excitability and behavioral outcome was examined in punished animals. There was no relationship between the average spikes evoked by 250 pA and pellets earned (*R*^2^ = 0.121, *P* = 0.652) or shocks delivered (*R*^2^ = 0.046, *P* = 0.786).

Learning-induced changes to the input–output relationship often result from a reduction in the spike frequency adaptation normally characteristic of BLA projection neurons and hippocampal pyramidal neurons (Motanis et al. 2014; Sehgal et al. 2014, 2023; Oh and Disterhoft 2015). To assess spike frequency adaptation, we examined the number of APs generated at 100 msec intervals (100 pA above rheobase [Fig. 2G]). As expected, the number of APs changed across the time intervals (*F*_(2.329,165.3)_ = 38.0, *P* < 0.0001), but no behavioral group difference (*F*_(3,70)_ = 2.45, *P* = 0.07) or interaction between behavioral group and time interval (*F*_15,355_ = 1.11, *P* = 0.35) emerged. Occasionally (∼10% of cases), the first and second APs occurred in rapid succession. It remains unclear whether this doublet reflects a distinct firing phenotype or represents a point along the continuum of spike frequency adaptation. Notably, the frequency of this firing pattern was similar across groups.

Intrinsic excitability is shaped by the expression and distribution of ion channels, which influence passive membrane properties, AP waveform characteristics, the frequency and timing of APs, and the afterhyperpolarization (AHP) that follows a burst of APs. We examined whether differences in these features underlie the observed excitability changes across behavioral groups. There were no group differences in the RMP, membrane time constant (τ), or Rm (Table 1).

**Table 1.** Biophysical properties

	Punished	Reward	Yoked	Naive	*F*	*P*
*n*	17	20	24	13		
RMP (mV)	−66.3 ± 1.1	−63.4 ± 1.4	−62.0 ± 1.0	−63.8 ± 1.4	1.99	0.12
Rm (MΩ)	85.5 ± 5.5	91.7 ± 4.9	90.1 ± 4.9	87.3 ± 6.5	0.780	0.51
Rs (MΩ)	11.3 ± 2.8	13.4 ± 3.0	11.5 ± 2.3	12.2 ± 3.4	0.644	0.59
τ (ms)	13.8 ± 0.7	13.6 ± 0.7	14.3 ± 0.6	14.4 ± 0.8	0.286	0.84
Thr. (mV)	−38.2 ± 1.5	−40.4 ± 1.0	−37.2 ± 0.9	−38.9 ± 9.0	2.64	0.056
AP_peak_ (mV)	76.6 ± 2.1	77.4 ± 1.6	75.6 ± 2.0	77.7 ± 1.9	1.40	0.25
AP_HW_ (ms)	1.11 ± 0.05	1.29 ± 0.02	1.10 ± 0.05	1.32 ± 0.06	3.91	0.012
fAHP (mV)	−8.5 ± 1.4	−4.9 ± 1.0	−7.5 ± 0.7	−6.1 ± 1.1	1.19	0.41
mAHP (mV)	−1.8 ± 0.4	−1.3 ± 0.3	−2.3 ± 0.4	−1.7 ± 0.5	1.39	0.25
I_AHP50_ (pA)	87.4 ± 10.4	42.1 ± 6.7	97.3 ± 9.1	68.9 ± 10.7	7.86	0.0001
I_AHP1000_ (pA)	11.1 ± 4.1	5.4 ± 2.0	15.2 ± 5.1	13.4 ± 3.8	1.16	0.33

Data are presented as mean ± SEM. (*n*) Number of cells per behavioral group; (RMP) resting membrane potential; (Rm) membrane resistance; (Rs) series resistance; (*t*) membrane time constant; (Thr.) action potential threshold; (AP_peak_) action potential amplitude; (AP_HW_) action potential half-width; (fAHP) fast hyperpolarization; (mAHP) medium afterhyperpolarization; (I_AHP50_) AHP Current 50 msec post-step; (I_AHP1000_) AHP current 1000 msec post-step.

We next used the first AP evoked at rheobase to the AP threshold, amplitude, half-width, and fAHP. One-way ANOVA showed the AP half-width varied between groups with post hoc analysis revealing that groups that received footshock (i.e., Punished, Yoked) had narrower APs than nonfootshock groups (i.e., Reward, Naive) (*F*_(1,70)_ = 11.3, *P* < 0.05; Fig. 3A,B). There was no difference between groups that had received footshock (i.e., Punished, Yoked) (*F*_(1,70)_ = 0.07, *P* > 0.05) or between nonfootshock groups (i.e., Reward, Naive) (*F*_(1,70)_ = 1.44, *P* > 0.05). No significant differences in AP half-width were observed between “learners” (those with *T*-ratios differences > 0) and “nonlearners” (unpaired *t*-test: *t*_(15)_ = 1.44, *P* = 0.13).

**Figure 3.**
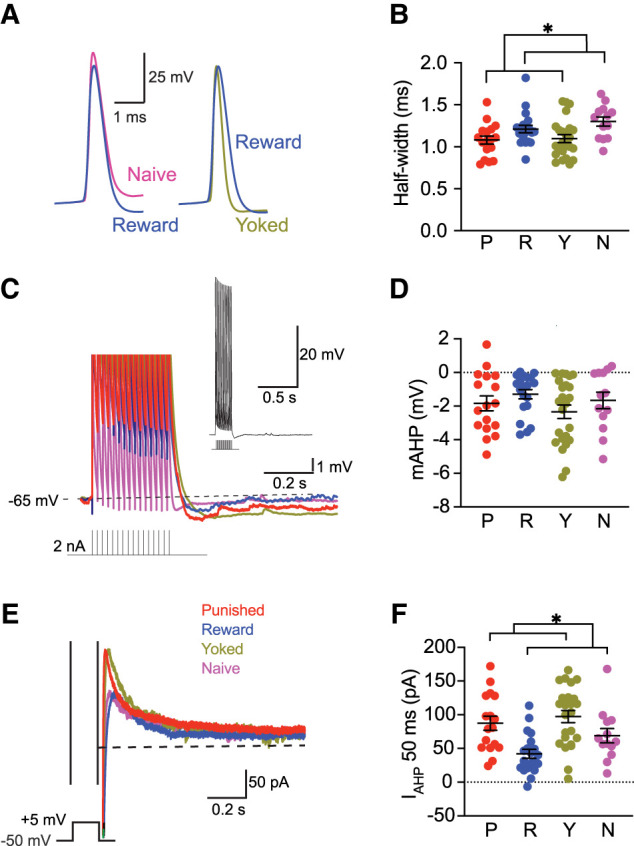
Effect of conditioning on biophysical properties. (*A*) Representative APs from Naive, Reward, and Yoked neurons. (*B*) APs from Punished and Yoked subjects were narrower than Reward and Naive. (*C*) Representative voltage response to a 300 msec 50 Hz train of 2 nA 2 msec current pulses is shown above (*inset*); the representative responses from Punished (P), Reward (R), Yoked (Y), and Naive (N) neurons are shown on an expanded voltage scale. (*D*) Summary data showing the peak mAHP. (*E*) Representative currents evoked by a 200 msec 55 mV voltage step. (*F*) Summary data showing the AHP current measured 50 msec after the voltage step (I_AHP50_ msec). The I_AHP50_ was larger in Punished and Yoked animals compared to Reward and Naive. (*) *P* < 0.05.

We did not resolve a difference in the amplitude of the postburst afterhyperpolarization (mAHP), following a 50 Hz train of 15 APs evoked by brief (2 msec 2 nA) somatic current injections (Fig. 3C,D). We then examined AHP currents under voltage clamp, holding cells at −50 mV and applying a 200 msec depolarizing voltage step to +5 mV (Fig. 3E,F). This protocol activates both the SK-mediated AHP current (I_AHP_) and the slower, apamin-insensitive sI_AHP_ (Power and Sah 2008; Power et al. 2011). Group differences were observed in the AHP current 50 msec after the voltage step (I_AHP50_) when both currents are active (*F*_(3,70)_ = 7.86, *P* = 0.0001), but not at 1 sec (*F*_(3,70)_ = 1.16, *P* = 0.33), when only the sI_AHP_ is active. Post hoc analysis revealed I_AHP50_ was larger in groups that received footshock (i.e., Punished, Yoked) than nonfootshocked groups (i.e., Reward, Naive) (*F*_(1,70)_ = 15.4, *P* < 0.05). No difference was found between the groups that received footshock (*F*_(1,70)_ = 0.63, *P* > 0.05) or between nonfootshock groups (*F*_(1,70)_ = 3.63, *P* > 0.05).

This study examined whether instrumental learning induces changes in BLA neuronal excitability similar to Pavlovian conditioning (Sehgal et al. 2014, 2023). Our findings extend prior work by showing that instrumental learning, both appetitive and aversive, increases excitability, which may facilitate integration of neurons into functional learning circuits to support synaptic integration and potentiation (Sah and Bekkers 1996; Power et al. 2011; Josselyn and Frankland 2018; Choi et al. 2021; Park et al. 2023). Together, these findings suggest that enhanced excitability is a core mechanism by which BLA neurons are functionally recruited during instrumental learning, potentially serving as a common cellular substrate for encoding both rewarding and aversive action–outcome associations.

Interpretation of punishment effects is confounded by punishment being layered on top of reward learning. While Punishment and Reward groups did not differ significantly in firing to current injections, the correlation between excitability and punishment performance, along with lower excitability in the Yoked group—which received an identical noncontingent shock exposure sequence—suggests that the instrumental punishment contingency plays a role in enhancing excitability. This finding raises the possibility that punishment-specific learning processes can preserve or augment the excitability increases initiated during reward training.

Surprisingly, intrinsic excitability was not enhanced in the Yoked group, which received noncontingent footshocks akin to Pavlovian fear conditioning, where excitability resembled that of naive controls and was lower than in both instrumental groups. This result contrasts prior studies showing fear-induced increases in BLA excitability (Sehgal et al. 2014, 2023). While those studies targeted neurons in the lateral amygdala, our recordings sampled from the basal nucleus, which has distinct projection targets and functional roles (Namburi et al. 2015; Beyeler et al. 2018). Furthermore, our task was conducted in a reward-associated context in the absence of an explicit conditioned stimulus. Supporting this finding, Butler et al. (2018) reported no change in intrinsic excitability following contextual fear conditioning when sampling broadly across the BLA. These results suggest that contextual fear conditioning, when superimposed on instrumental training or delivered in a minimally salient context, may not engage the same excitability-enhancing mechanisms observed in classical cued fear paradigms.

We observed group differences in AP half-width and current underlying the post-burst AHP, though their relationship to excitability changes remains unclear. Narrower APs and larger I_AHP50_s were observed in shock-exposed animals (Punished and Yoked) compared to nonshocked animals (Reward and Naive), whereas excitability was enhanced in the Reward and Punished groups. In BLA projection neurons, narrower APs (Faber and Sah 2002) and larger AHPs are associated with reduced excitability (Faber and Sah 2002; Power and Sah 2008). Thus, these changes may reflect a distinct biophysical adaptation linked to shock exposure rather than learning. These analyses were conducted post hoc and should be interpreted cautiously; future studies using targeted designs and more sensitive electrophysiological methods will be essential to confirm these effects and clarify their mechanisms.

We did not resolve differences in the post-burst AHP, a major contributor to spike frequency adaptation that is often reduced following learning a variety of tasks in the amygdala and elsewhere (McKay et al. 2013; Sehgal et al. 2014; Titley et al. 2020). This may result from the small amplitude and thus limited resolution of our recorded AHPs, likely resulting from use of gluconate rather than MeSO_4_ as the primary internal solution anion (Zhang et al. 1994; Kaczorowski et al. 2007).

In summary, instrumental punishment learning enhances BLA excitability comparable to reward learning, while delivery of aversive stimuli in a noncontingent manner suppresses these increases. These results suggest that punishment and aversive Pavlovian associations may be encoded differently by BLA neurons. However, aversive stimuli also induced distinct biophysical changes independent of learning contingencies. These findings may inform our understanding of psychiatric disorders involving altered punishment sensitivity, such as depression and conduct disorder, where maladaptive behaviors persist despite negative consequences.
